# Identification of hypoglycemia-specific neural signals by decoding murine vagus nerve activity

**DOI:** 10.1186/s42234-019-0025-z

**Published:** 2019-07-11

**Authors:** Emily Battinelli Masi, Todd Levy, Tea Tsaava, Chad E. Bouton, Kevin J. Tracey, Sangeeta S. Chavan, Theodoros P. Zanos

**Affiliations:** 1 Zucker School of Medicine at Hofstra/Northwell, Heampstead, NY USA; 2 Institute of Bioelectronic Medicine, Feinstein Institute for Medical Research, Manhasset, NY 11030 USA

**Keywords:** Hypoglycemia, Decoding, Vagus nerve, Insulin, Glucose, Bioelectronic medicine

## Abstract

**Background:**

Glucose is a crucial energy source. In humans, it is the primary sugar for high energy demanding cells in brain, muscle and peripheral neurons. Deviations of blood glucose levels from normal levels for an extended period of time is dangerous or even fatal, so regulation of blood glucose levels is a biological imperative. The vagus nerve, comprised of sensory and motor fibres, provides a major anatomical substrate for regulating metabolism. While prior studies have implicated the vagus nerve in the neurometabolic interface, its specific role in either the afferent or efferent arc of this reflex remains elusive.

**Methods:**

Here we use recently developed methods to isolate and decode specific neural signals acquired from the surface of the vagus nerve in BALB/c wild type mice to identify those that respond robustly to hypoglycemia. We also attempted to decode neural signals related to hyperglycemia. In addition to wild type mice, we analyzed the responses to acute hypo- and hyperglycemia in transient receptor potential cation channel subfamily V member 1 (TRPV1) cell depleted mice. The decoding algorithm uses neural signals as input and reconstructs blood glucose levels.

**Results:**

Our algorithm was able to reconstruct the blood glucose levels with high accuracy (median error 18.6 mg/dl). Hyperglycemia did not induce robust vagus nerve responses, and deletion of TRPV1 nociceptors attenuated the hypoglycemia-dependent vagus nerve signals.

**Conclusion:**

These results provide insight to the sensory vagal signaling that encodes hypoglycemic states and suggest a method to measure blood glucose levels by decoding nerve signals.

**Trial registration:**

Not applicable.

## Background

Evolution conferred organisms with reflexes that maintain physiological homeostasis by continuously monitoring and effectively modulating organ function. Among the different types of neuronal reflexes that can monitor and respond to changes in critical biomarkers, the neuronal circuitry that maintains glucose homeostasis is of specific importance and interest. Glucose is the primary energy source for mammalian cells, however deviations of circulating blood glucose levels outside of the normal range can have detrimental effects. Hypoglycemia in particular, either insulin-induced due to diabetes or other less common causes such as medication side effects or hormone deficiencies, can be even more detrimental, leading to loss of consciousness, seizures, coma and even death (Marks 1972). The brain is critically dependent on glucose since it cannot synthesize or store significant amounts of blood glucose (Cryer et al. 2003). The central nervous system has been implicated in glucose homeostasis (Verberne et al. 2014) via the mechanisms that maintain blood glucose levels within a narrow healthy range.

Early studies revealed several central nervous system mechanisms for sensing glucose and hypoglycemic states of the organism (Bernard 1855; Masi et al. 2018; Schwartz and Porte 2005). Neurons in the septum, amygdala, striatum, motor cortex, hindbrain, and hypothalamus (McCrimmon 2008) increase firing rates when blood glucose levels drop, and activity is inhibited when plasma glucose levels rise (Routh 2002). Changes in peripheral glucose levels lead to release of insulin and leptin in the hypothalamus (Inoue 2015; Obici et al. 2002; Schwartz et al. 2013).

The peripheral nervous system is involved in relaying changes in glucose levels to the brain to maintain glucose homeostasis (Verberne et al. 2014; Masi et al. 2018; Kaelberer et al. 2018). Glucose sensing neurons are present in pancreatic beta cells, the intestine, the hepatic portal vein, the carotid body (McCrimmon 2008) or the gut (Fournel et al. 2016). Signaling from these sensors is carried to various brain regions through peripheral nerves, mainly the vagus nerve and its major brainstem nuclei (Fournel et al. 2016; Berthoud 2008; Jordan et al. 2010; Niijima 1989). Afferent discharges of the hepatic branch of the vagus significantly decrease following the administration of D-glucose (Niijima 1982), and the brain-liver circuit that runs through the vagus has been shown to be required to restrain glucose production in the presence of insulin (Pocai et al. 2005). Neurophysiological recordings have also revealed the role of the vagus nerve and its branches in relaying sensory information about the metabolic state of the animal. Recorded activity from afferent fibers of the hepatic branch of the vagus nerve increases after portal vein glucose injections in a glucose dose-dependent manner (Niijima 1984). Type 2 diabetic rats have decreased vagus neuron activation in response to intestinal glucose (Lee et al. 2012). The vagus nerve integrates other important metabolic signals related to the microbiota (Bonaz et al. 2018), nutrient sensory transduction (Niijima 1984) and gut induced reward (Han et al. 2018). Although it has been suggested that this afferent nerve signalling may be part of a metabolic reflex to hypoglycemia (Lee and Miller 1985), until now, no studies have examined neural responses in the vagus nerve to insulin-triggered hypoglycemia.

We recently developed a framework to isolate and decode the neural activity recorded on the surface of the vagus nerve in mice to identify groups of neurons firing in response to specific cytokines (Steinberg et al. 2016; Zanos 2019; Zanos et al. 2018). Here, we recorded vagal responses specific to hypoglycemia and correlated this activity with circulating blood glucose levels in a mouse model. We observed that neural signals in the vagus nerve respond significantly to insulin-induced hypoglycemia and correlate with dropping blood glucose levels.

## Methods

### Electrophysiological recordings and experimental design

#### Animals

All experimental protocols were approved by the Institutional Animal Care and Use Committee at the Feinstein Institute for Medical Research, Northwell Health, which follows National Institutes of Health guidelines for the ethical treatment of animals. Male BALB/c were purchased from Charles River Laboratory. Animals were used between the ages of 8–12 weeks, weight 20–26 g unless otherwise noted. Animals were housed at 25 °C, with ad libitum water and chow, and acclimated to a 12 h light and dark cycle for at least 1 week prior to conducting experiments.

#### Recording procedure

Mice were fasted for 3–4 h prior to each experiment. They were induced with 2.5% isoflurane and maintained at 1.7%. Mice were placed in the supine position, and a midline cervical incision was made. The left cervical vagus nerve was isolated from the carotid bundle. The extraneural sheath overlaying the nerve was removed to ensure good contact with the leads, and the nerve was placed in a cuff electrode with a 200 μm inner diameter (PDMS Sling μCuff, CorTec). The neural recording was acquired using Plexon Omniplex Neural Data Acquisition system, with a sampling rate of 40 kHz. A ground electrode was placed next to the right salivary gland. The nerve was kept from desiccation by placing parafilm over the recording area. Following acquisition of baseline activity for 30 min, animals were treated with insulin (6 mg/kg Humulin-R U-100, Eli Lilly Medical) or glucose (2 g/kg) intraperitoneally (i.p.), and recordings were continued for 30 min post-injection. Glucose measurements were taken before and throughout the recording via tail nick and Abbott Freestyle glucometer.

### Data analysis

The signal processing framework from our previous work (Zanos et al. 2018) conditions the raw vagal neurograms and extracts information from them in order to correlate the neural firing rates with the blood glucose level. This framework first removes the electrocardiogram artifacts using wavelets to isolate the compound action potentials (CAPs) that are detected using an adaptive threshold. Dimensionality reduction is applied to the detected CAP waveforms and the results are clustered to represent groups of fibers that fire together. Finally, the event rates of each cluster are derived from the data points in each cluster.

#### Determining responsive neural groups

Only a fraction of the fibers in the vagus are expected to transmit signals related to changes in metabolic state. Furthermore, we only record from a fraction of the fibers, so there is no guarantee that the recording will contain signals from responsive fibers. A detected response occurs when one or more of the recorded CAP clusters exhibits a change in firing statistics in reaction to a change in the metabolic state induced by an injection. We determine which datasets exhibit a response and present our results using only these recordings.

Changes in firing rate are determined via an optimization procedure that doesn’t make any assumptions about the distribution of firing rates and is invariant across CAP groups and recordings. We measure the distribution of firing rates for each CAP before and after the injection for each injection type including the control group where we inject saline. A threshold is swept across the pre-injection firing rates that extends from t = 10 min to t = 30 min. At each threshold position, any data points with a firing rate that exceeds the threshold is labeled a false alarm. Any data point from the post-injection distribution that exceeds the same threshold is labeled a detection. The post-injection window extends from t = 40 min to t = 60 min. The first 10 min are discarded because of potential transient responses and artifacts. This process is repeated separately for left and right sided thresholds because excitatory or inhibitory circuits could be involved, and the side with the maximum probability of detection is selected.

We want to find the left tail and right tail thresholds that maximize the number of responsive insulin and glucose injection datasets subject to allowing one responsive saline dataset or two responsive TRPV1 saline datasets. By inspection, one of the saline datasets and two of the TRPV1 saline datasets show a visible response in the firing rate to injection. A responsive dataset is one that has a probability of detection above the probability of detection threshold that is determined by allowing one or two saline responses at the optimal left and right tail false alarm rate thresholds.

#### Neural decoding

To assess whether neural responses to insulin injections can be used to estimate the blood glucose concentration of the subject, regression models were trained using recordings that showed a response to the injection. The features used as input to the regression models are the means of the non-overlapping sliding windows of each CAP event rate. The windows were chosen to be 20 s long and go back in time for 4 min. The features were regressed against the spline-interpolated smoothed blood glucose measurements without a time-shift.

To avoid overfitting, lasso regularization with a regularization factor of 1 as well as 12-fold leave-one-out cross-validation were used. A separate model was trained for each fold and the out-of-sample data was evaluated on the model. Twelve folds was selected because selecting too few folds won’t adequately capture the entire input space, and too many folds would more strongly violate the assumption that the training set and test set were independently generated. A diagram of the neural decoder is shown in Fig. 3.

The mean absolute error is computed by averaging the absolute value of the residuals from the smoothing-spline-interpolated blood glucose concentration measurements and the reconstructed blood glucose concentration from the out-of-sample data.

## Results

### Surface vagus nerve recordings respond to insulin-induced hypoglycemia

**Fig. 1:**
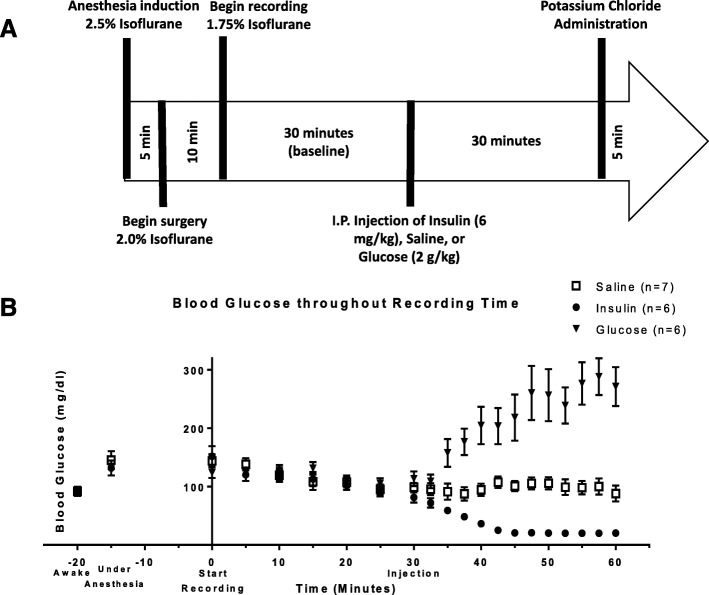
Recording Neural Events in Acute Hyper and Hypoglycemia. **a** Mice are anesthetized with isoflurane, the nerve is fitted with a bipolar, cuff electrode from Cortec and activity is recorded using a Plexon acquisition system. After 30 min’ baseline recording, mice received 6 mg/kg insulin, 2 g/kg glucose, or saline, and were recorded for an additional 30 min. **b** Blood glucose was measured via tail nick with glucometer throughout the recording. Blood glucose dropped significantly in mice that received insulin within 10 min. Blood glucose rose significantly in mice that received glucose

**Fig. 2:**
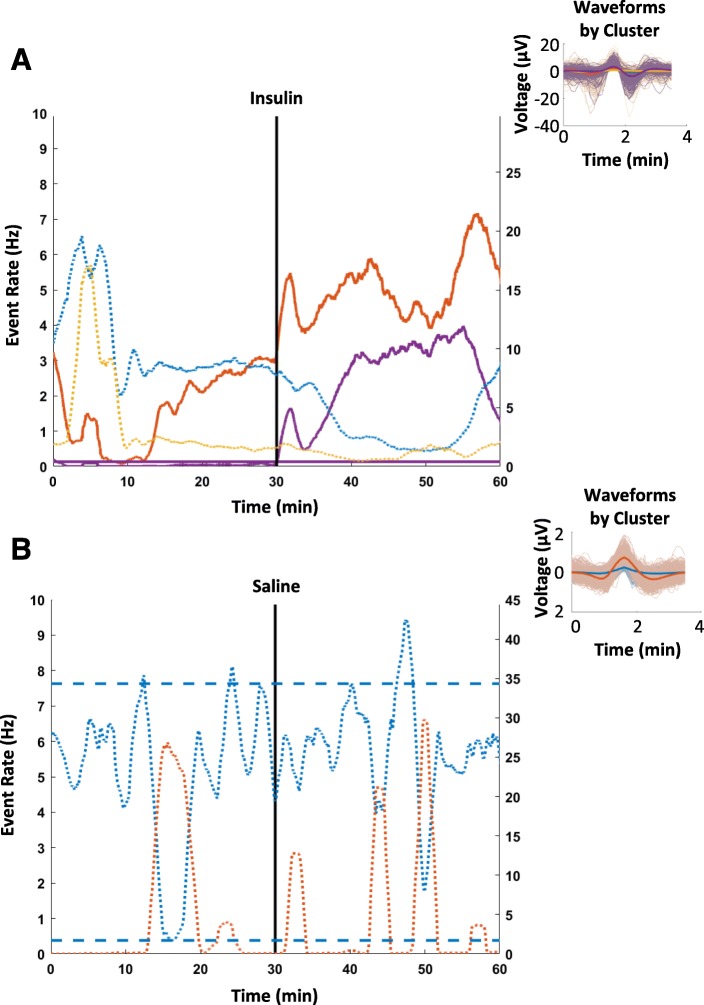
Indicative neural responses to different injections. Each colored trace represents the response rate over time of a different CAP – solid lines correspond to lower firing rate CAPs (maximum of 10 CAPs/sec) and dotted lines corresponds to high firing rate CAPs (maximum of 30–45 CAPs/sec). The injection is indicated by a vertical black line occurring at 30 min. The thresholds encapsulating the baseline statistics of a particular CAP are indicated by horizontal lines of the same color. An example of **a** insulin VN response curves that include a CAP that increases its firing rate, along with their respective CAP waveforms and **b** an example of neural responses to the saline injections control, where there are no discernible responses to the injection, along with the respective CAP waveforms.

We recorded from the surface of the vagus nerve in anesthetized mice (*N* = 19) using a Cortec Micro Cuff Sling electrode during acute hypoglycemia induced by insulin injections (Fig. 1a and b). After 30 min of baseline recordings, mice received 6 mg/kg insulin, 2 g/kg glucose, or saline, and activity was recorded for 30 additional minutes. Blood glucose levels were measured via tail nicks and the use of a standard glucometer at regular time intervals (every 5 min before injection and every 2.5 min after injection). Measured blood glucose levels dropped significantly (4.1 mg/dl/min over 15 min) in mice that received insulin and rose significantly (5.3 mg/dl/min over 30 min) in mice that received glucose (Fig. 1b). Using our previously established approach recording afferent neural activity (Zanos et al. 2018; Silverman et al. 2018), we analyzed afferent responses to changes in circulating blood glucose levels. CAPs, a signature of synchronous activation of groups of fibers, were detected using our previous pre-processing framework (Zanos et al. 2018). For each experiment, the animal was defined as a “responder” when the pre-injection and post-injection firing rate distributions were significantly different for at least one CAP group (Table 1). We observed that the vast majority of these recordings exhibited a change in the firing rate of these CAPs after the injection of insulin, with a response rate of 83.3%. These changes manifested mainly as an increase in the firing rate of CAPs (Fig. 2a, 80% of responders). In our control experiments of saline injections, no discernible change in the firing rate of the detected CAPs was observed (Fig. 2b, response rate of 14.3%).

**Table 1:** Population results of the detected responders on insulin and saline injection experiments. Insulin injections elicited a response 83.3% of the time and tended to cause an increased rate of firing. Saline injections elicited a response 14.3% of the time

Injection Type	Percent Responders	Increased Firing	Decreased Firing
Insulin (*N*=6)	83.3%	80.0%	20.0%
Saline (*N*=7)	14.3%	100.0%	0.0%

### Vagus responses to hypoglycemia correlate with blood glucose levels

**Fig. 3:**
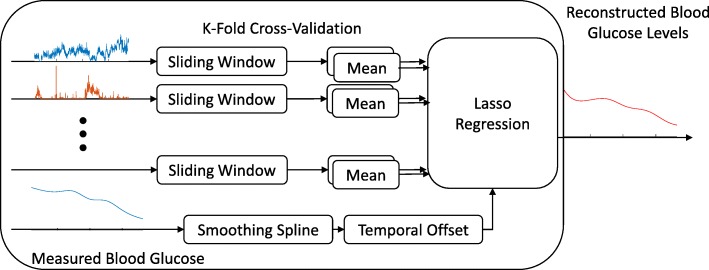
Decoding Algorithm and illustrative example. Schematic Diagram of the decoder used to regress the CAP event rates to the blood glucose concentration. The means of 20 s, non-overlapping sliding windows over the event rates for the previous 4 min were used as features to the lasso regression model with a regularization factor of 1. The inputs were regressed against a non-time-shifted version of the smoothing spline fit to the measurements of the blood glucose concentration. Leave-one-out cross-validation with 12 folds was used to train 12 separate regression models, and the out-of-sample data was evaluated for each model to reconstruct a piecewise response

**Fig. 4:**
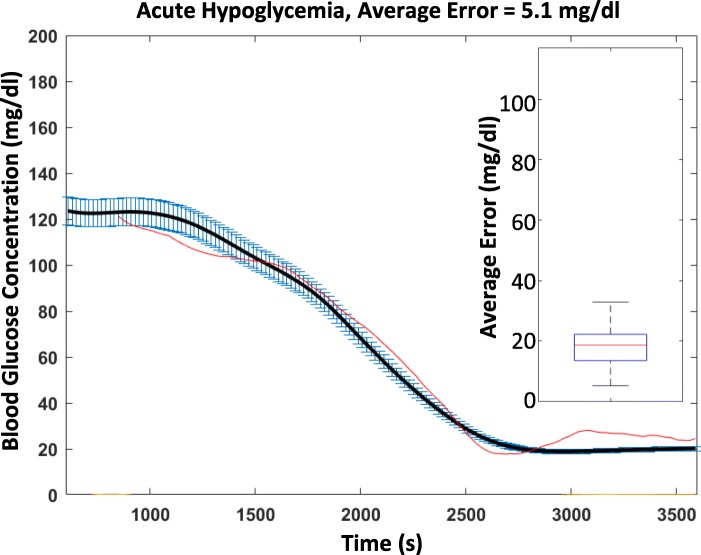
Indicative wild type regression model performance from an insulin injection. The black line indicates the smoothed blood glucose measurements, with blue bars indicating interpolation error. The red line indicates the estimated blood glucose levels as an output of the regression model. The regression closely traces the smoothed blood glucose measurements, and the average error over the *N* = 6 subjects has an interquartile range between 14 and 22 mg/dl

While these results show that the vagus nerve responds to acute hypoglycemia, it is not clear whether information about the actual blood glucose levels is also relayed through the same conduit. To examine this, we regressed the firing rates of CAPs to the measured blood glucose levels by performing linear regression with regularization (Methods, Fig. 3). We quantified the ability of our regression model to estimate blood glucose levels from neural activity by computing the average error of the estimation relative to the measured blood glucose. In the example plotted in Fig. 4, the estimated blood glucose levels from our regression model have values close to the measured blood glucose levels (average error of 5.2 mg/dl). Across all hypoglycemia experiments, the regression algorithm maintained its accuracy with a median average error of 18.6 mg/dl (Fig. 4).

### Vagus nerve recordings from TPRV1-DTA mice contain a subset of neural responses to insulin injections

**Fig. 5:**
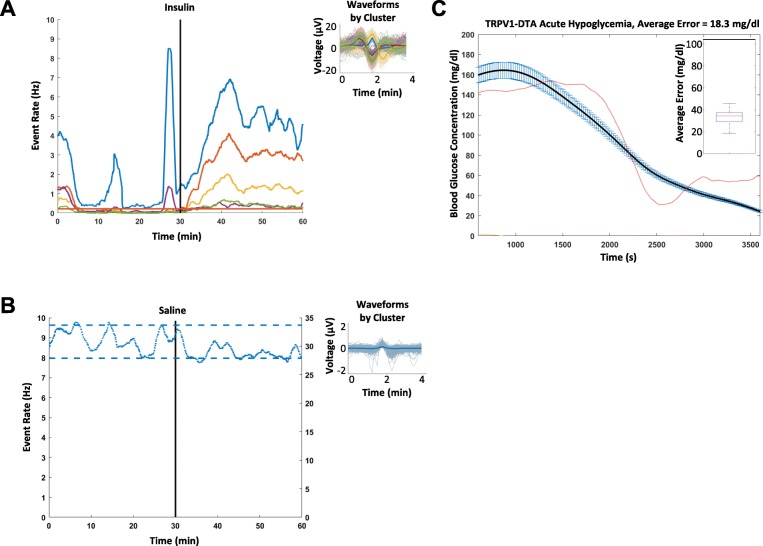
Indicative neural responses to different injections from TRPV1+ Cell-depleted mice. Each colored trace represents the response rate against time of a different CAP – solid lines correspond to lower firing rate CAPs (maximum of 10 CAPs/sec) and dotted lines corresponds to high firing rate CAPs (maximum of 30–50 CAPs/sec). The injection is indicated by a vertical black line occurring at 30 min. The thresholds encapsulating the baseline statistics of a particular CAP are indicated by horizontal lines of the same color. An example of the **a** insulin VN response curves, along with their respective CAP waveforms, and **b** an example of the neural response to the saline injection control, where there is no discernible response to the injection, along with the respective CAP waveform. An example of TRPV1+ Cell-depleted mice **c** regression model performance from an insulin injection. The black line indicates the smoothed blood glucose measurements, with blue bars indicating measurement error. The red line indicates the estimated blood glucose levels as an output of the regression model. The regression somewhat traces the smoothed blood glucose measurements, and the average error over the *N* = 7 subjects has an interquartile range between 29 and 37 mg/dl

We have shown previously that TRPV1 fibers are necessary for cytokine-specific sensory signaling through the vagus nerve (Zanos et al. 2018). Since vagus nerve activity increased as a response to hypoglycemia, we reasoned that this response may be mediated by TRPV1 fibers. Accordingly, we administered insulin in TRPV1 cell depleted mice (Table 2, *N* = 7) while recording from the vagus nerve. A smaller percentage of the recordings showed a response to insulin (57.1%) as compared with the wild type mice (83.3%), but insulin still caused an increase (75% of responders) in firing rates (Fig. 5a). Only a small percent of the saline administered TRPV1 cell-depleted mice elicited an afferent vagal response (Fig. 5b, response rate of 33.3%, *N* = 6), akin to wild type mice. Since vagus nerve responses of TRPV1 cell depleted mice to insulin injections were reduced, we hypothesized that blood glucose level related information included in these responses would be compromised. As shown in the example of Fig. 5c, the regression model performed worse but maintained some accuracy (average error of 18.3 mg/dl). Across all TRPV1 cell-depleted mice hypoglycemia experiments, our decoding algorithm performed significantly worse than the wild type counterparts (one-tailed, two-sample t-test, *p* = 0.006), with a median average error of 34.3 mg/dl (Fig. 5c insert).

**Table 2:** Population results of the detected responders on all injection experiments for TRPV1 cell depleted mice. Insulin injections elicited a response 57.1% of the time and tended to cause an increased rate of firing. Saline injections elicited a response 33.3% of the time

Injection Type	Percent Responders	Increased Firing	Decreased Firing
TRPV1-DTA Insulin (*N*=7)	57.1%	75.0%	25.0%
TRPV1-DTA Saline (*N*=6)	33.3%	100.0%	0.0%

### Vagus nerve activity does not respond consistently to glucose injection induced hyperglycemia

**Fig. 6:**
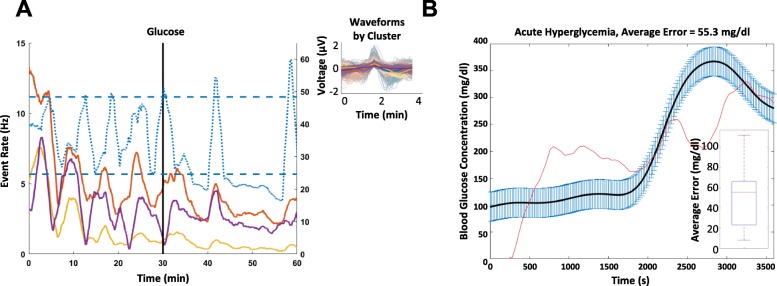
An example of neural responses to a glucose injection. Each colored trace represents the response rate against time of a different CAP – solid lines correspond to lower firing rate CAPs (maximum of 15 CAPs/sec) and dotted lines corresponds to high firing rate CAPs (maximum of 60 CAPs/sec). The injection is indicated by a vertical black line occurring at 30 min. The thresholds encapsulating the baseline statistics of a particular CAP are indicated by horizontal lines of the same color. The **a** glucose VN response curves include a CAP that decreases its firing rate are shown here along with their respective waveforms. An example of wild type **b** regression model performance from a glucose injection. The black line indicates the smoothed blood glucose measurements, with blue bars indicating interpolation error. The red line indicates the estimated blood glucose levels as an output of the regression model. The regression revolves around the smoothed blood glucose measurements, and the average error over the *N* = 6 subjects has an interquartile range between 25 and 66 mg/dl

The vagus nerve responds to low blood glucose levels, however we wanted to examine whether this signaling is specific to hypoglycemia and not hyperglycemia. Because of the inverse correlation between blood glucose levels and vagus nerve activity shown from previous studies and our results above, it is expected that hyperglycemic conditions would result in lower firing rates. We induced hyperglycemia by administering glucose (Fig. 1a and b), while recording from the vagus nerve (Table 3, *N* = 6). After glucose injections, the firing rates decreased in some cases (60% of responders, Fig. 6a) and increased in others (40% of responders). Moreover, the correlation between the firing rates and the blood glucose concentrations became weaker relative to the correlations during hypoglycemia, with the median average error for the hyperglycemia experiments being 55.6 mg/dl, more than 3 times higher than the hypoglycemia experiments (Fig. 6b). Together these results indicate that sensory vagal signaling encodes hypoglycemic states in a more consistent manner than hyperglycemic states and suggest a method to measure blood glucose levels by decoding nerve signals.

**Table 3:** Population results of the detected responders on glucose injection experiments. Glucose injections also elicited a response 83.3% of the time and tended to cause a decreased rate of firing

Injection Type	Percent Responders	Increased Firing	Decreased Firing
Glucose (*N*=6)	83.3%	40.0%	60.0%

## Discussion

In this study, we have found that signals from the surface of the cervical vagus nerve change with acute hypoglycemia, and that some of the signals are correlated with blood glucose levels. Prior studies have reported elevated brain activity in the hypothalamic, as well as other regions, in response to acute hypoglycemia (McCrimmon 2008; Routh 2002; Zhou et al. 2018). While some mechanisms for sensing this decrease in blood glucose occur within the central nervous system (Saberi et al. 2008), the peripheral signals involved in our analysis are likely to contribute to this activity because the vagus nerve has been implicated as the main conduit of the brain-liver circuit, required to restrain glucose production in the presence of insulin (Pocai et al. 2005). However, the vagal sensory arm of this circuit was not previously examined. The afferent fibers from the hepatic branch of the vagus nerve that respond to the blood glucose concentration in the hepatic portal vein transmit neural signals that pass through the cervical level of the vagus (Niijima 1984). These studies showed decreased activity of these fibers due to hyperglycemia, revealing the anti-correlation between blood glucose levels and vagus nerve activity. Our results confirm this anti-correlation by establishing for the first time elevated nerve activity due to hypoglycemia. Our experiments do not show conclusive results regarding neural responses to hyperglycemia. However these responses were highly variable, partly due to the small number of animals. Similar future experiments with a larger number of animals could uncover responses not visible in our current cohort. Beyond the vagus nerve, recent studies have shown that other peripheral nerves, such as the carotid sinus nerve and the carotid bodies might also sense blood glucose and insulin levels (Koyama et al. 2000; Limberg et al. 2014; Ribeiro et al. 2013; Wehrwein et al. 2010). Moreover, glucose uptake and restriction has a direct effect on the sympathetic nervous system by modulating its activity (Rowe et al. 1981).

The specific fibers and channels involved in the generation of this neural signaling are not yet identified but nociceptors, including TRPV1, could be prime candidates in relaying these signals. TRPV1 is a cation channel that responds to certain noxious stimuli like capsaicin and is mainly expressed in afferent neuronal cell bodies in the dorsal root, trigeminal and nodose/jugular ganglia as well as adjacent brain nuclei (Caterina et al. 1997; Story et al. 2003). Prior work determined that TRPV1 fibers were necessary for cytokine mediated responses (Zanos et al. 2018). TRPV1 has also been implicated in dysregulation of metabolic and specifically glucose homeostasis (Derbenev and Zsombok 2016; Gram et al. 2017; Suri and Szallasi 2008). However, whether TRPV1 fibers are not only necessary, but also sufficient for responses to hypoglycemia has not been examined previously. Based on our results, this pathway conveys some of this information but is not the only population necessary for this communication. Since TRPV1 channels have been shown to be necessary and sufficient for cytokine signaling in the vagus nerve (Zanos et al. 2018), our results show that on top of TRPV1, a different group of neurons is involved in a significant part of this signaling. A candidate subtype of neurons that could be implicated in this signaling is transient receptor potential cation channel subfamily A member 1 (TRPA1) (Derbenev and Zsombok 2016) due to its role in the vago-vagal reflex that modulates the outflow to the subdiaphragmatic organs, as well as its involvement in the response to oxidative stress that can contribute to metabolic dysfunction. While a large percentage of TRPA1 channels co-localize with TRPV1 channels, a significant 10% of non-respiratory TRPV1- vagal afferents are TRPA1+ (Nassenstein et al. 2008), supporting that the activity correlated with blood glucose in TRPV1 cell depleted mice could be carried by TRPA1+ afferents. Na_V_1.7 could be another candidate subtype for relaying these signals. The majority of vagal afferent fibers contain Na_V_1.7 channels (Muroi et al. 2011), and other recent work has implicated Na_V_1.7 and Na_V_1.8 in colon projection dorsal root ganglion neurons with colonic hypersensitivity in diabetic rats (Hu et al. 2016). Further studies into the populations of neurons carrying this information, as well as their possible functional connectivity patterns as these relate to certain states (Zanos et al. 2011) would provide valuable advances in the understanding of the mechanism of this sensing.

In this study, all the recordings were acute and the experiments were terminal. It will be informative to use vagus nerve recordings in chronically implanted awake animals with implantable telemetry to measure blood glucose levels in real time. Chronic models would enable the recording of activity without the confounding effects of anesthesia on neuronal signaling. Chronic experiments would also yield a higher number of detectable features, such as the derivative of the glucose level, and possibly enable the development of algorithms with lower blood glucose reconstruction errors. Nevertheless, this proof-of-concept study that for the first time correlates blood glucose levels to afferent vagus signaling showcases the feasibility of a neural based glucose measurement device.

The current results lay the groundwork for developing a closed-loop recording and stimulating device which could simultaneously monitor and intervene to maintain proper glucose homeostasis in a patient with dysregulation of this process. Bioelectronic estimates of blood glucose levels from the cervical vagus would have several benefits over current methods for measuring blood glucose concentration. Use of glucometers through finger pricks require frequent human intervention and are subject to issues of patient compliance. Artificial pancreas devices that consist of glucose sensors and insulin pumps have a sensor life that spans from a couple weeks (Klonoff 2007) to 6 months (Aronson et al. 2018) and also require replacing the insulin. Stimulation of various nerves could be an alternative to injection of insulin as a way to modulate blood glucose levels. Low frequency stimulation on the cervical vagus nerve has been shown to increase blood glucose in rats and selective efferent and afferent stimulation on the same target has been shown to differentially affect insulin and blood glucose (Meyers et al., 2016). The efferent hepatic vagus has also been implicated in the control of hepatic glucose production (Kimura et al., 2016; Matsuhisa et al., 2000). In humans, vagus nerve high frequency electrical block was used as a therapy over the course of a year and led to a clinically relevant 1 % reduction in glycosylated hemoglobin in diabetic patients (Shikora et al., 2013). A fully closed-loop device would deliver the necessary stimulation to regulate blood glucose levels only when needed and present a novel long-term treatment plan for hypo- or hyperglycemic patients.

## Conclusion

The current studies demonstrate that the vagus nerve carries important signals from the periphery to the central nervous system regarding glucose homeostasis. Further understanding of this neural code offers new possibilities for diabetes management and therapy.

## Data Availability

Please contact authors for data requests.

## Abbreviations

CAP: Compound action potential
DTA: Diphtheria toxin fragment A
i.p.: intraperitoneally
TRPA1: Transient receptor potential cation channel subfamily A member 1
